# The clue is on the lip: unmasking esophageal adenocarcinoma: A case report

**DOI:** 10.1097/MD.0000000000042837

**Published:** 2025-06-20

**Authors:** Abuoma Cherry Ekpendu, Muhammad Sohaib Asghar, Gregory May, Thomas Jewell, Pankajkumar Patel

**Affiliations:** a Department of Internal Medicine, AdventHealth Sebring, Sebring, FL; b Department of Pathology, AdventHealth Sebring, Sebring, FL; c Department of Gastroenterology, AdventHealth Sebring, Sebring, FL.

**Keywords:** adenocarcinoma, esophageal cancer, esophagogastroduodenoscopy, lip lesions

## Abstract

**Rationale:**

Esophageal cancer accounts for about 1% of all cancer diagnoses in the United States.

**Patient concerns:**

An 82-year-old male presented to a dermatology clinic with a chief complaint of a skin lesion on the right lower lip. He denied any prior gastrointestinal symptoms or history.

**Diagnoses:**

A shave biopsy of the lip lesion revealed adenocarcinoma. Subsequent positron emission tomography–computed tomography demonstrated findings concerning for esophageal malignancy with metastatic disease.

**Interventions:**

Esophagogastroduodenoscopy identified a lesion in the distal esophagus, and biopsy confirmed invasive, moderately to poorly differentiated esophageal adenocarcinoma.

**Outcomes:**

The patient was referred to oncology, where he was diagnosed with stage IV metastatic esophageal adenocarcinoma. He was initiated on chemotherapy, which he tolerated well.

**Lessons:**

This case highlights the rare presentation of stage IV esophageal adenocarcinoma as a solitary lip lesion without typical gastrointestinal symptoms. Given the rarity of lip involvement, clinicians should maintain a high index of suspicion for systemic malignancy in atypical skin lesions. Early biopsy and diagnosis are crucial for timely intervention and improved outcomes. The purpose of the study is to highlight the importance of maintaining a high index of suspicion for gastrointestinal malignancy, even in the absence of traditional gastrointestinal symptoms, when evaluating unusual skin lesions.

## 1. Introduction

Esophageal cancer accounts for approximately 1% of all cancer diagnoses in the United States, though its prevalence is higher in certain regions, including northern China, Southeast Asia (such as India), southern Africa, and Iran.^[1]^ In 2024, an estimated 22,370 new cases of esophageal cancer were diagnosed, with approximately 16,130 deaths attributed to the disease. Among these, 17,690 new cases and 12,880 deaths occurred in men, while 4680 new cases and 3250 deaths were reported in women. Esophageal adenocarcinoma (EAC) and esophageal squamous cell carcinoma are the 2 most common histological subtypes of esophageal cancer.

The incidence of esophageal cancer varies by race and ethnicity. It is more common in the White population, where EAC is the predominant histologic subtype. In contrast, squamous cell carcinoma is more prevalent among African Americans. American Indians, Alaska Natives, and Hispanics have lower rates of esophageal cancer, while the lowest incidence is observed among Asians and Pacific Islanders.^[1]^ Most patients with EAC present at an advanced stage, contributing to poor prognosis.^[2]^

Reports of EAC metastasizing to the lip are exceedingly rare. This case report aims to highlight the rare and unique presentation of Stage IV EAC manifesting as a solitary lip lesion, in the absence of typical gastrointestinal symptoms. It underscores the need for clinicians to maintain a high index of suspicion for systemic malignancy when evaluating atypical skin lesions. Additionally, it raises awareness of this uncommon metastatic site and contributes to the broader understanding of esophageal cancer’s diverse metastatic patterns.

## 2. Case presentation

**Figure 1.**
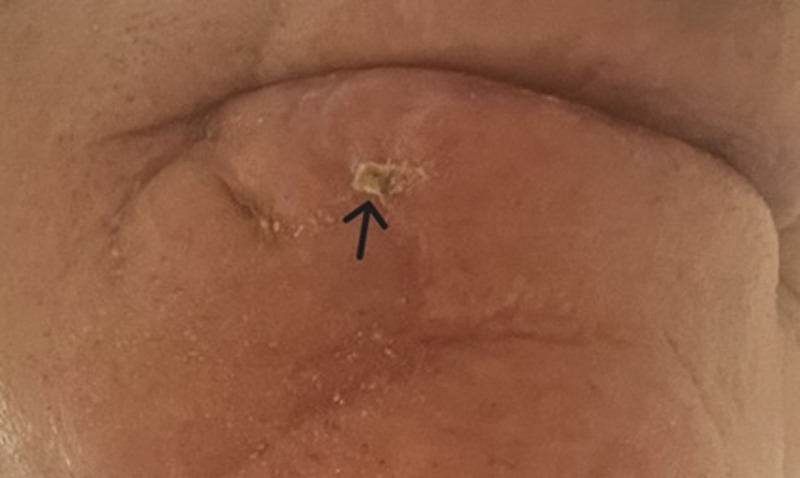
Shows the lip lesion on the right lower cutaneous lip.

An 82-year-old male presented to a dermatology clinic with a chief complaint of a mildly painful skin lesion on the right lower lip (Fig. 1). The lesion had been noted approximately 1 month prior to presentation. The patient reported a previous medical history significant for skin cancers including basal cell carcinoma, squamous cell carcinoma, and lentiginous melanoma, all of which had been managed by dermatology. He also had a family history of colon cancer.

At the time of presentation, the patient denied experiencing any gastrointestinal symptoms, including dysphagia, heartburn, early satiety, nausea, vomiting, bloating, or abdominal pain. Additionally, he reported no recent weight loss, hoarseness, or loss of appetite. His most recent colonoscopy, performed 5 years prior, identified colon polyps, but he had never undergone an esophagogastroduodenoscopy (EGD). The patient was a lifetime nonsmoker and consumed alcohol infrequently.

Physical examination revealed a papular, flesh-colored, firm lesion on the right lower cutaneous lip, approximately 0.6 cm in size, circumscribed with well-defined borders. The remainder of the examination, including the head, neck, chest, abdomen, back, and extremities, was unremarkable. The initial differential diagnosis included squamous cell carcinoma, basal cell carcinoma, benign neoplasm, and infection.

A shave biopsy was performed in the office and biopsy results revealed adenocarcinoma. To investigate the primary source of malignancy, the patient underwent a positron emission tomography-computed tomography (PET/CT) scan, which revealed mural thickening and significant glucose hypermetabolism of the distal esophagus, gastroesophageal junction, and proximal stomach. Additionally, the scan showed extensive hematogenous metastatic disease to the liver, as well as multifocal musculoskeletal metastatic involvement, including 4 vertebrae, the left clavicle, pelvic bones, right infratemporal fossa pterygoid, left deltoid, right shoulder girdle muscles, and metastatic disease to the liver, gastrohepatic ligament, celiac nodes, and pulmonary hilar nodes.

**Figure 2.**
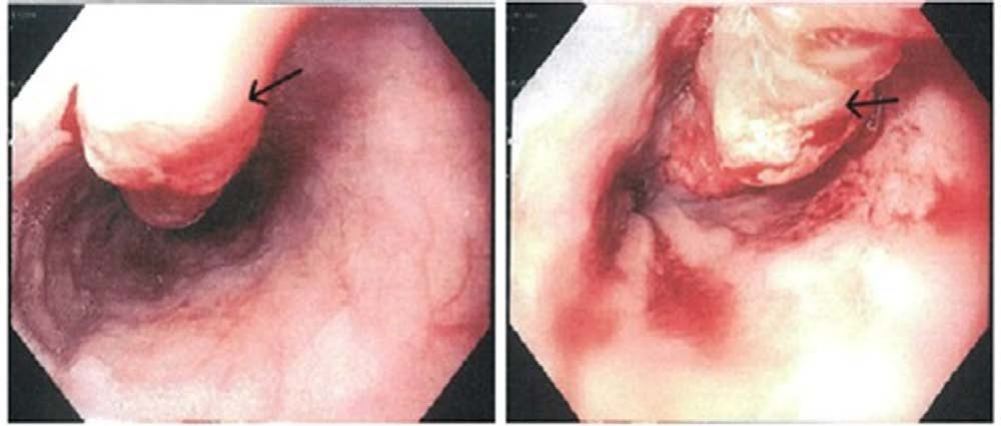
Endoscopy image shows the 8-cm-long lesion in the distal esophagus.

**Figure 3.**
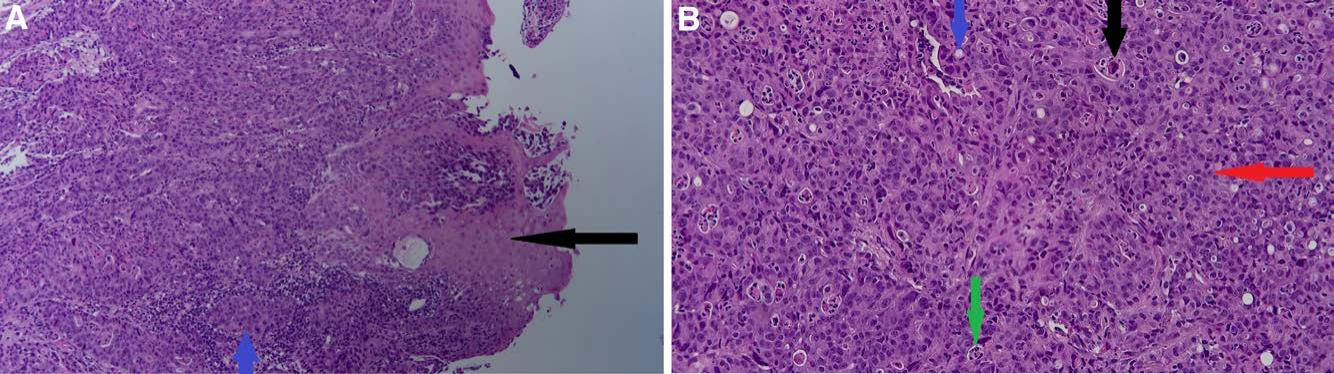
(A) Histology (low power field) reveals the esophageal adenocarcinoma (blue arrow) and esophageal squamous mucosa (black arrow). (B) Histology (high-power field) reveals the esophageal adenocarcinoma on a high-power field; including the typical neoplastic cellular process seen in adenocarcinoma including anaplastic nucleoli (red arrow), rudimentary gland formation (blue arrow), necrotic tumor cells (black arrow), micro abscess (green arrow).

The patient was then referred to gastroenterology for EGD with biopsy. Laboratory studies revealed a normal white blood cell count of 6.34 × 10³/µL (microliter) and a hemoglobin level of 11.6 g/dL (gram per deciliter), with an MCV of 93.5 fL (femtoliter), indicating normocytic anemia. However, given the absence of symptoms or active bleeding, no outpatient treatment was pursued. Liver function tests were within normal limits, with AST at 12 U/L (units per liter), ALT at 10 U/L, and total bilirubin at 0.24 mg/dL (milligram per deciliter). Renal function was also normal, with a creatinine level of 0.92 mg/dL and an estimated glomerular filtration rate (eGFR) of 83.1 mL/min (milliliters per minute). EGD identified an 8 cm lesion in the distal esophagus extending into the gastroesophageal junction (Fig. 2). A biopsy of the lesion was performed, and histopathology confirmed invasive, moderate to poorly differentiated EAC. No evidence of Barrett esophagus was noted (Fig. 3).

Subsequently, the patient was referred to oncology for further evaluation and treatment, where he was diagnosed with Stage IV EAC. He received radiation therapy and was also initiated on chemotherapy with FOLFOX (leucovorin calcium, fluorouracil, and oxaliplatin). He had a brief hospitalization for anemia and dark stools after initiating outpatient chemotherapy – during which his hemoglobin level dropped to 6.4 g/dL, for which he received packed red blood cells transfusion. He was not a good candidate for upper endoscopy per gastroenterology recommendations. He was hemodynamically stable throughout his hospital course and his hemoglobin levels improved to 7.8 g/dL and the dark stools resolved. The patient was discharged in stable condition after 3 days of hospital stay. Outpatient follow-up with the oncology team revealed that patient remained stable on FOLFOX.

## 3. Discussion

The incidence of esophageal cancer in the United States is 4.2 per 100,000 individuals, with an estimated 51,185 prevalent cases. In 2024, 22,370 new cases were diagnosed, making esophageal cancer the 17th most common malignancy in the U.S. It is most frequently diagnosed in individuals aged 65 to 74, with a median age at diagnosis of 68, and is more common in males.^[3]^

Risk factors for EAC include gastroesophageal reflux disease (GERD), Barrett esophagus, high body mass index, and low socioeconomic status. The diagnosis of esophageal cancer should be considered in the presence of clinical features such as dysphagia, weight loss, and fatigue. Upper gastrointestinal endoscopy (EGD) is the diagnostic modality of choice, allowing for direct visualization and biopsy. Once EAC is diagnosed, a PET/CT scan is recommended to evaluate for distant metastasis. Endoscopic ultrasound is the standard for locoregional staging and, if necessary, for obtaining biopsy specimens from suspicious lymph nodes. For patients with neurological symptoms, MRI of the brain is recommended to assess for metastasis. Advanced lesions, particularly those involving the middle third of the esophagus, may invade the trachea, and bronchoscopy may be performed to assess for such involvement. Individuals with Barrett esophagus require regular EGD monitoring due to their increased risk of EAC.^[4,5]^

The treatment of EAC depends on tumor stage, location, and the patient’s comorbidities. Over the past decade, endoscopic therapy for superficially invasive EACs <3 cm has evolved. Initial treatment typically involves endoscopic resection, which provides staging information. Complete resection is followed by endoscopic eradication therapy to remove any residual Barrett esophagus. Radiofrequency ablation is the most commonly used modality for eradication. Due to the risk of local recurrence, surveillance endoscopy is recommended every 3 months during the first year post-therapy, every 4 to 6 months during the second year, and annually thereafter. Esophagectomy is reserved for patients with poor esophageal function or those with superficial lesions, though this surgery carries significant morbidity.^[4,5]^

For locally advanced disease, the optimal approach remains unclear, despite numerous studies. Neoadjuvant chemotherapy and external beam radiotherapy are commonly used in the U.S. before surgery. Esophagectomy is an important treatment option for locally advanced disease, with various surgical approaches described. Minimally invasive esophagectomy is the most recently developed approach. Mortality remains high despite neoadjuvant therapy, with 3-year overall survival ranging from 32% to 59% and median survival between 16 and 49 months for patients receiving chemoradiation therapy. Ongoing research aims to identify novel chemotherapeutic agents. For patients with metastatic or unresectable disease, chemotherapy or chemoradiation remains the standard treatment. Palliative endoscopic therapies, such as dilation to alleviate dysphagia, may be used. Tumor debulking procedures are also employed to improve quality of life.^[4,5]^

A systematic review of esophageal cancer metastasis revealed that the head and neck were the most common unexpected metastasis sites with about 66% of esophageal cancer metastasis to this region originating from the lower esophagus as the initial tumor site. Whereas adenocarcinoma, comprised 50% of cases metastasizing to the skin and muscle.^[6]^

While the percentage of lip lesions representing metastasis is unclear, 1% to 8% of all oral tumors represent malignant metastasis from different types of cancers with the most common location being the mandible, gingiva, maxilla and tongue in that order. Notably 1.4% of esophageal cancers overall metastasize to the oral cavity, with 83.3% to the jaw bones and 16.7 to the soft tissues.^[7]^

Literature review reveals that skin metastasis from esophageal carcinoma is more commonly seen in older males with cases typically presenting with symptoms such as dysphagia, regurgitation, weight loss, and loss of appetite.^[8–11]^ However, 1 case described an otherwise healthy patient who presented with a small, painless, mobile facial skin nodule, which was later confirmed as metastatic adenocarcinoma. Further investigations revealed the primary esophageal tumor, despite the absence of systemic symptoms.^[12]^

While metastasis from esophageal cancer to the skin has been documented,^[8–11]^ reports of EAC metastasizing specifically to the lip are exceedingly rare. In contrast, the case presented here is unique in that the solitary lip lesion was the only presenting sign, with no accompanying symptoms or prior history of dysphagia, GERD, or gastrointestinal malignancy.

Lip lesions can present with diverse morphologies, including papules, plaques, patches, ulcers, and nodular growths, which may obscure an underlying systemic malignancy. This constitutes a diagnostic challenge as varied presentation of lip lesions can contribute to misdiagnosis and delayed recognition of underlying systemic disease, emphasizing the need for a thorough evaluation in atypical cases.^[13]^

This case report is inherently limited by its nature as a single-patient observation, which restricts the generalizability of its findings. Additionally, the absence of long-term follow-up data prevents an assessment of treatment response, disease progression, and overall prognosis. Future research should focus on larger case series to better characterize the incidence and clinical features of EAC metastasizing to the lip. Molecular and histopathological analyses could provide further insights into the metastatic mechanisms and pathways involved in this rare presentation.

Our case highlights the rare and unique presentation of Stage IV EAC as a solitary lip lesion, without the typical accompanying symptoms such as dysphagia, GERD, or weight loss. While metastasis to the skin from esophageal cancer is well documented, lip involvement is exceedingly rare. This underscores the importance of maintaining a high index of suspicion for systemic malignancy, even in the absence of traditional gastrointestinal symptoms, when evaluating unusual skin lesions. Early recognition and biopsy of atypical skin lesions can facilitate timely diagnosis of underlying malignancies and significantly impact patient outcomes. Clinicians should be aware that EAC may present in unexpected ways, and prompt evaluation and treatment are crucial for improving survival in patients with advanced disease.

## Author contributions

**Conceptualization:** Abuoma Cherry Ekpendu, Gregory May.

**Data curation:** Abuoma Cherry Ekpendu.

**Project administration:** Pankajkumar Patel.

**Software:** Muhammad Sohaib Asghar.

**Supervision:** Pankajkumar Patel.

**Validation:** Abuoma Cherry Ekpendu, Muhammad Sohaib Asghar, Gregory May.

**Visualization:** Abuoma Cherry Ekpendu, Muhammad Sohaib Asghar, Thomas Jewell.

**Writing – original draft:** Abuoma Cherry Ekpendu.

**Writing – review & editing:** Abuoma Cherry Ekpendu, Muhammad Sohaib Asghar, Thomas Jewell.
